# The Association of Left Ventricular Hypertrophy with Metabolic Syndrome is Dependent on Body Mass Index in Hypertensive Overweight or Obese Patients

**DOI:** 10.1371/journal.pone.0016630

**Published:** 2011-01-31

**Authors:** Federico Guerra, Lucia Mancinelli, Luca Angelini, Marco Fortunati, Alessandro Rappelli, Paolo Dessì-Fulgheri, Riccardo Sarzani

**Affiliations:** Department of Internal Medicine and Cardiovascular Diseases, “Hypertension Excellence Centre” of the European Society of Hypertension, University Hospital “Ospedali Riuniti,” University “Politecnica delle Marche,” Ancona, Italy; University of Tor Vergata, Italy

## Abstract

**Background:**

Overweight (Ow) and obesity (Ob) influence blood pressure (BP) and left ventricular hypertrophy (LVH). It is unclear whether the presence of metabolic syndrome (MetS) independently affects echocardiographic parameters in hypertension.

**Methods:**

380 Ow/Ob essential hypertensive patients (age ≤65 years) presenting for referred BP control-related problems. MetS was defined according to NCEP III/ATP with AHA modifications and LVH as LVM/h^2.7^ ≥49.2 g/m^2.7^ in males and ≥46.7 g/m^2.7^ in females. Treatment intensity score (TIS) was used to control for BP treatment as previously reported.

**Results:**

Hypertensive patients with MetS had significantly higher BMI, systolic and mean BP, interventricular septum and relative wall thickness and lower ejection fraction than those without MetS. LVM/h^2.7^ was significantly higher in MetS patients (59.14±14.97 vs. 55.33±14.69 g/m^2.7^; p = 0.022). Hypertensive patients with MetS had a 2.3-fold higher risk to have LVH/h^2.7^ after adjustment for age, SBP and TIS (OR 2.34; 95%CI 1.40–3.92; p = 0.001), but MetS lost its independent relationship with LVH when BMI was included in the model.

**Conclusions:**

In Ow/Ob hypertensive patients MetS maintains its role of risk factor for LVH independently of age, SBP, and TIS, resulting in a useful predictor of target organ damage in clinical practice. However, MetS loses its independent relationship when BMI is taken into account, suggesting that the effects on MetS on LV parameters are mainly driven by the degree of adiposity.

## Introduction

Obesity and obesity-related hypertension are rapidly increasing worldwide together with their metabolic and cardiovascular complications [1]–[3]. Left ventricular hypertrophy (LVH) is one of the complications and is, in turn, an important risk factor for myocardial infarction, heart failure, stroke, and cardiac sudden death [4]. Blood pressure (BP) is the main determinant of the hemodynamic workload for the left ventricle and, in turn, of left ventricular mass (LVM) [5], although both hemodynamic and non-hemodynamic factors are involved in the complex pathogenesis of LVH [6], [7]. Obesity represents both a hemodynamic and non-hemodynamic risk factor for LVH, even independently of BP [7], and increasing body mass index (BMI), the most studied and commonly used in practice index of adiposity, is by itself associated with increasing cardiovascular and metabolic complications [1].

The metabolic syndrome (MetS), an insulin-resistant state characterized by a cluster of cardiovascular risk factors, is increasing in prevalence in developed and developing countries too [8]. MetS is strongly associated with increased risk for both type 2 diabetes and cardiovascular disease [9]–[11], although criticisms have been raised about its role as independent risk factor beyond the contribution of each of its components [12]–[14].

The main aim of this study was to evaluate whether MetS is an independent risk factor for LVH in overweight/obese (Ow/Ob), non-elderly, hypertensive patients. In particular, we aimed to verify whether the relationship between MetS and LVH is independent from BMI.

## Methods

In this cross-sectional study, 436 consecutive patients referred to our Hypertension Centre from January 2006 to April 2009 because of BP-control-related problems were evaluated. Inclusion criteria were: a) essential untreated hypertension or stable anti-hypertensive drug treatment during the previous 6 months; b) Ow/Ob (BMI≥25 kg/m^2^). When clinically indicated [15], a complete study to exclude secondary hypertension was performed. Exclusion criteria were: age older than 65 years (to reduce age-related overlapping and confounding factors such as increasing prevalence in vascular and renal damage), low compliance to anti-hypertensive drug therapy as investigated by Morisky Medical Adherence Scale (MMAS) [16] to evaluate adherence levels, severe renal damage defined as glomerular filtration rate (eGFR) <30 ml/min, diabetes mellitus type 1, any race other than white Caucasian, heart failure NYHA III or IV or left ventricular ejection fraction (LVEF) <50%, liver failure, cancer or other systemic severe diseases. Patients with incomplete clinical or echocardiographic data were also excluded. After this selection, 380 patients met the inclusion and exclusion criteria and were enrolled in the study. Each participant gave informed written consent and all clinical investigations have been conducted according to the principles expressed in the Declaration of Helsinki. This observational study was approved by local institutional ethics committee (Comitato Etico, Azienda Ospedali Riuniti, Ancona).

### Measurements

Body weight and height were measured on a standard beam balance scale with an attached ruler. Body weight was measured to the nearest 0.1 kg, and height was measured to the nearest 1 cm. Waist circumference was measured in orthostatism with the patient standing relaxed, arms freely hanging at each side, and feet close together by using a flexible plastic tape to the nearest 1 cm according and classified to NECP ATP III [17].

BP was measured following indications of the ESH-ESC guidelines [15] using validated mercury-free digital sphygmomanometers (A&D, UM-101) with appropriate cuff size. The average of three consecutive measurements was used for the analysis. Controlled BP was defined as systolic BP (SBP) <140 mmHg and diastolic BP (DBP) <90 mmHg. When type 2 diabetes was present, values <130/80 mmHg were used as cut-offs to define controlled BP. In a subset of patients (n = 184), when clinically indicated following ESH guidelines [15], 24-hour ambulatory blood pressure measurements (Spacelabs, 90207) were also taken and analyzed.

Blood samples for plasma fasting glucose, total and HDL cholesterol, triglycerides, and creatinine as well as first morning urine specimens to determine albumin creatinine ratio (ACR) were obtained. All analyses were performed in the certified (ISO 9001∶2000) University Hospital Central Laboratory. Microalbuminuria was defined as ACR ≥22 mg/g of urinary creatinine in men and ≥31 mg/g in women [15]. Glomerular filtration rate (eGFR) was estimated by using the Modification of Diet in Renal Disease Study equation [18].

MetS was defined according to NCEP/ATP III classification as modified by the AHA [17], when, in addition to high BP (which was an inclusion criteria and therefore a common feature of all enrolled patients), two or more of the following criteria were also present: waist ≥102 cm in men and ≥88 cm in women, HDL ≤40 mg/dl in men and ≤50 mg/dl in women, triglycerides ≥150 mg/dl, and fasting glucose ≥100 mg/dl (or diagnosis of type 2 diabetes).

### Anti-hypertensive treatment

To allow for comparability of drug regimens across patients taking many different medications, a treatment intensity score (TIS) was calculated. As previously reported [19], the recorded daily dose taken by the patient was divided by the maximum recommended daily dose to obtain a proportional dose for that medication, called intensity. For completeness, dual-class drugs were separated into their components and intensities were calculated separately for each of the chemical compounds. Maximum recommended daily doses set by the Italian national agency for drugs (Agenzia Italiana del Farmaco, AIFA) at the time of each single visit were used for calculations. The sum of all the different values was recorded as TIS.

### Echocardiography

Left ventricular dimensions were measured by echocardiography (ATL HDI 5000, Philips) following the American Society of Echocardiography recommendations [20]. For each patient the following measurements were taken: end-diastolic and end-systolic interventricular septum thickness (IVSD and IVSS, respectively), posterior wall thickness (PWD and PWS, respectively), and left ventricular diameters (LVDD and LVDS, respectively); left atrial diameter (LAD). LVM was calculated (M-mode tracings under two-dimensional control, left parasternal short axis view, mean of three cardiac cycles) by using the Devereux's formula [21] and indexed by either body surface area (LVMi) or height^2.7^ (LVM/h^2.7^) [22]. Because all of the patients were overweight or obese, LVH was defined on the basis of the LVH/h^2.7^, using ≥49.2 g/m^2.7^ in men and ≥46.7 g/m^2.7^ in women as partition values [23]. Myocardial relative wall thickness (RWT) was also calculated and a RWT≥0.45 defined concentric remodeling (CR) [24]. LVEF was calculated as (LV end-diastolic area – LV end-systolic area)/LV end-diastolic area (two dimensional apical four-chambers view, mean of three cardiac cycles) [25].

### Statistical Analysis

The study was planned to have a sample size of ≥120 subjects in each group. The sample size was calculated on averages and standard deviations of previous publications exploring similar issues [26]. This study had a >80% power to detect a LVM/h^2.7^ difference ≥2.5 g/m^2.7^ between patients with or without MetS (with α = 0.05), assuming a standard deviation of 11 g/m^2.7^. Differences between patients with or without MetS were evaluated by using analysis of variance (ANOVA) adjusted for age and sex for continuous variables and the χ^2^ test for categorical variables. Logistic regression analysis was used to create adjusted models including independent variables associated with LVH/h^2.7^. SPSS 13.0 for Windows (SPSS Inc. Chicago, IL, USA) was used for all the statistical analyses. A value of p<0.05 was considered as statistically significant.

## Results

Clinical characteristics of the 380 studied patients, adjusted for age and sex, are shown in Table 1. Prevalence of MetS in our sample was 65% despite age <65 years old. Three hundred thirty seven patients (88.7%) were on stable treatment for at least 6 months whereas forty three (11.3%) were untreated. 86 patients (23.9%) had diabetes mellitus type 2. Among treated patients, no differences were found in TIS and in prevalence of each single anti-hypertensive drug class between patients with and without MetS. Among many expected differences, MetS patients had significantly higher BMI, eGFR and ACR.

**Table 1:** General characteristics of the population.

Variable	Ow/Ob hypertensives(n = 380)	No MetS(n = 133)	MetS(n = 247)	p
Sex (M/F)	251/129	85/48	166/81	.52
Age (yrs)	52.4±9.1	52.1 (0.8)	52.9 (0.6)	.42
Hypertension diagnosis (yrs)	5.4±7.1	5.7 (0.7)	5.5 (0.7)	.82
Anti-hypertensive therapy (%) †	88.7	88.7	88.6	.93
Dyslipidemia (%) †	77.1	63.1	84.6	**<.001**
Diabetes (%) †	23.9	0.9	32.0	**<.001**
Lipid-lowering therapy (%) †	15.8	0.0	24.3	**<.001**
Hypoglycemic therapy (%) †	9.7	2.2	13.8	**<.001**
Smoking habit (%) †	52.1	48.1	54.2	.25
BMI (kg/m^2^)	31.97±5.41	30.9 (0.5)	33.3 (0.4)	**<.001**
Waist (cm)	107.82±13.36	102.9 (1.2)	109.6 (1.1)	**.001**
SBP (mmHg)	150.24±19.46	145.6 (1.7)	152.2 (1.3)	**.002**
DBP (mmHg)	90.92±12.63	89.1 (1.1)	91.5 (0.8)	.08
MBP (mmHg)	110.69±13.08	137.6 (1.5)	142.3 (1.1)	**.014**
Fasting glucose (mg/dl)	106.98±35.85	98.1 (3.1)	126.6 (2.3)	**.007**
Total cholesterol (mg/dl)	205.80±47.10	204.1 (4.3)	207.2 (3.7)	.59
HDL (mg/dl)	43.93±11.23	51.0 (0.9)	42.6 (0.8)	**<.001**
Triglycerides (mg/dl)	173.86±146.93	110.9 (12.5)	212.0 (10.9)	**<.001**
LDL (mg/dl)	127.07±39.73	131.2 (3.6)	122.2 (3.1)	.06
GFR (ml/min)	103.63±30.45	97.9 (2.6)	105.4 (1.9)	**.023**
ACR (mg/g creat)	57.60±203.09	17.2 (26.7)	67.2 (19.7)	**.035**
TIS	1.49±1.05	1.45 (0.10)	1.56 (0.07)	.36

Results of analysis of variance (ANOVA);

† results of χ^2^ test. Data are mean ± SD or absolute numbers. Data, adjusted for age and sex, are expressed as mean (standard error). Fasting glucose is adjusted for age, sex and hypoglycemic therapy. Total cholesterol, HDL, triglycerides and LDL are adjusted for age, sex and lipid-lowering therapy.

Echocardiographic parameters, adjusted for age and sex, are shown in Table 2. LVMi was not significantly different between Ow/Ob hypertensive patients with or without MetS, whereas those with MetS had significantly higher LVM/h^2.7^ than those without. MetS patients had also significantly higher RWT and lower LVEF.

**Table 2:** Echocardiographic characteristics of the population.

Variable	Ow/Ob hypertensivesn = 380	No MetSn = 133	MetSn = 247	p
IVSTD (mm)	10.51±1.56	10.10±1.39	10.49±1.63	**.019**
IVSTS (mm)	15.01±2.25	14.77±2.38	14.88±2.18	.702
PWTD (mm)	9.88±1.40	9.56±1.36	9.82±1.41	.079
PWTS (mm)	15.50±2.14	15.43±2.01	15.32±2.21	.703
LVIDD (mm)	52.02±5.51	51.17±5.03	51.47±5.76	.617
LVIDS (mm)	33.80±5.94	31.87±5.60	33.04±5.94	.069
LAD (mm)	40.22±5.58	39.55±6.07	39.86±5.30	.616
LVMi (g/m^2^)	116.19±30.16	111.17±30.19	113.28±30.14	.506
LVM/h^2.7^ (g/m^2.7^)	58.28±14.99	55.33±14.69	59.14±14.97	**.022**
RWT	.39±.06	.38±.05	.40±.06	**.024**
LVEF (%)	65.74±10.11	67.99±9.97	65.51±10.07	**.028**

Results of analysis of variance (ANOVA). Data are mean ± SD; data adjusted for age and sex, are expressed as mean (standard error).

In univariate analysis, MetS was strictly related to the presence of cardiac hypertrophy as defined by LVH/h^2.7^ and CR. Patients with MetS had a 2.8-fold higher relative risk to have CR (OR 2.81 95% CI 1.41–5.62; p = 0.002) and a 2.3-fold higher relative risk to have LVH/h^2.7^ (OR 2.28; 95% CI 1.43–3.62; p<0.001). When MetS was present in Ow/Ob hypertensive patients, prevalence of CR raised from 8.3% to 20.2% while prevalence of LVH/h^2.7^ increased from 61.7% to 78.5% (see IC and p values above).

Logistic regression models were used to test the independent role of risk factors for LVH/h^2.7^. In the first model including each single criteria of MetS (SBP, DBP, waist, fasting glucose, HDL cholesterol and tryglicerides along with diagnosis of diabetes or dyslipidemia), only SBP resulted independently associated with LVH/h^2.7^ (table 3, model 1). When BMI was introduced in the model (instead of waist) both SBP and BMI resulted as independent risk factors (table 3, model 2). In another model including SBP, MetS, therapy (as described by TIS) and BMI all of them except MetS resulted as independent risk factors for LVH/h^2.7^ (Table 4, model 1). However, once BMI was excluded from the model, MetS resulted significantly associated to LVH/h^2.7^ (Table 4, model 2). The inclusion in the latter models of dichotomous variables representing current active treatment with common anti-hypertensive drug classes (i.e. ACE-I/ARBs, β-blockers, calcium channel blockers) did not affect the results. In the subgroup of patients (n = 184, 48%) in which 24-hour ambulatory blood pressure measurements were available, the use of 24-hour SBP instead of “office” SBP confirmed the results, and the overall fit of the models actually improved (Nagelkerke square 0.32, table 4, model 3 and 4).

**Table 3:** Independent risk factors for LVH/h^2.7^ assessed by logistic regression models.

Variable	Model 1				Model 2			
	OR	95% CI		p	OR	95% CI		p
Waist	1.02	0.99	1.02	.099				
SBP	1.03	1.01	1.06	.002	1.03	1.01	1.05	<.001
DBP	1.01	0.97	1.03	.381	1.02	0.99	1.03	.193
Fasting glucose	1.01	0.99	1.03	.222	1.00	0.99	1.01	.953
HDL	0.97	0.94	1.00	.054	0.98	0.96	1.01	.165
Triglycerides	1.00	0.99	1.00	.938	1.00	0.99	1.00	.214
Diabetes	0.28	0.22	1.53	.276	1.01	0.44	2.32	.971
Dyslipidemia	0.77	0.42	1.91	.772	0.82	0.44	1.53	.538
BMI					1.22	1.13	1.31	<.001

Model 1 included waist, SBP, DBP, fasting glucose, HDL cholesterol and triglycerides along with diagnosis of diabetes or dyslipidemia as covariates. Model 2 included all model 1 variables except waist, which was substituted by BMI, as covariates. No adjustment for sex was applied because of the different partition values for LVH/h^2.7^ used for males and females.

**Table 4:** Independent risk factors for LVH/h^2.7^ assessed by logistic regression models.

Variable	Model 1				Model 2			
	OR	95% CI		p	OR	95% CI		p
SBP	1.02	1.00	1.03	.009	1.02	1.00	1.03	.010
MetS	1.68	0.97	2.92	.065	2.34	1.40	3.92	.001
TIS	1.36	1.02	1.80	.036	1.46	1.12	1.92	.006
BMI	1.19	1.10	1.28	<.001				

Variable	Model 3				Model 4			
	OR	95% CI		p	OR	95% CI		p
24-h SBP	1.06	1.03	1.09	.006	1.05	1.02	1.08	.001
MetS	2.74	0.96	6.99	.061	4.62	2.15	9.94	<.001
TIS	1.77	1.14	2.63	.010	1.85	1.26	2.76	.002
BMI	1.18	1.06	1.33	<.001				

Model 1 included SBP, MetS, TIS and BMI as covariates. Model 2 included all model 1 variables except BMI as covariates. Model 3 and 4 are similar respectively to model 1 and 2 but with 24-hour SBP instead of ambulatory SBP. No adjustment for sex was applied because of the different partition values for LVH/h^2.7^ used for males and females.

Regarding gender, both in men and in women MetS is associated with a similar increase in LVH/h^2.7^ prevalence (from 60% to 78% in men and from 68% to 79% in women) and with a similar relative risk for LVH/h^2.7^ (OR 2.1 for women and 2.4 for men; all p<0.05). Unfortunately, the study sample size was not planned to test gender-dependent differences in MetS and LVH relationship. Moreover, different partition values for LVH were used in men and women.

**Figure 1:**
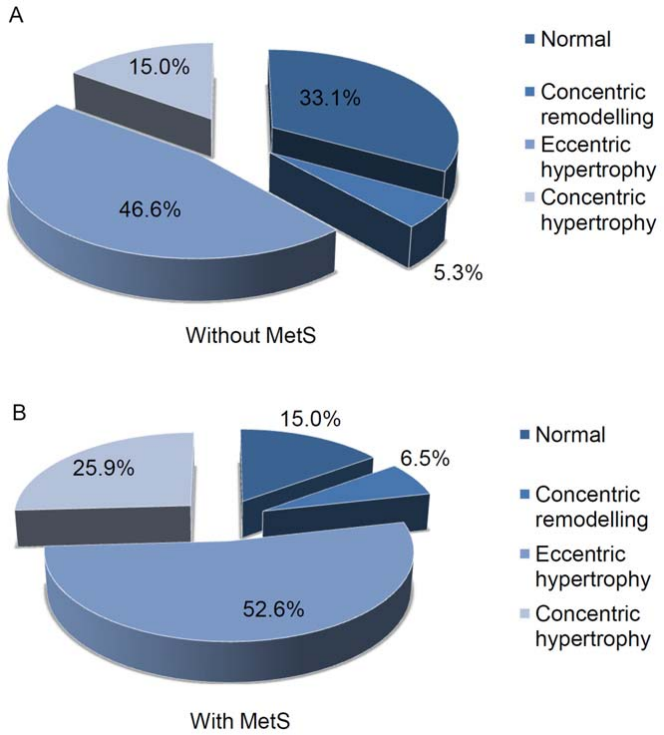
Prevalence of concentric remodelling, eccentric and concentric hypertrophy in Ow/Ob hypertensive patients.

Left ventricles without both CR and LVH/h^2.7^ were defined as “normal”, LV with CR in the absence of LVH/h^2.7^ were defined as having LV “concentric remodeling”, LV with LVH/h^2.7^ but without CR were defined as having “eccentric hypertrophy”, and LV with both CR and LVH/h^2.7^ were defined as having “concentric hypertrophy”. Their respective prevalences with or without MetS are shown Figure 1. Distribution of these subtypes of cardiac damage between patients with or without MetS were significantly different (χ^2^ test, p<0.001), with MetS patients having higher prevalence of CR, eccentric hypertrophy, and concentric hypertrophy (Figure 1).

## Discussion

Ow/Ob hypertensive patients often have high cardiovascular risk and the presence of LVH and/or MetS further increase their risk. It is unclear whether MetS is an independent risk factor for LVH in these patients and the present study aimed at investigating this issue. The main finding of our investigation was that in Ow/Ob, non elderly, hypertensive patients MetS is not associated with LVH/h^2.7^ when BMI is taken into account.

The “surprising” finding that BMI is the driving factor behind MetS-related LV increased mass was not totally unexpected but has never been specifically tested before, especially in a context of ow/ob non-elderly hypertensive patients.

We chose to investigate LVH as defined by LVM/h^2.7^ cut-offs because body surface area correction reduces variability due to body size and gender [27] and underestimates LVM in the upper range of the body surface area distribution [22]. Thus, normalizing by height^2.7^ seems to offer the most accurate estimation of LVM and risk factors for pathologic changes in heart structure in overweight and obese subjects [28]. Moreover, even direct unadjusted measurements of cardiac remodeling such as IVSTD and RWT, showed similar differences between patients with and without MetS.

It has been previously demonstrated that in females MetS had a greater impact on LVH and the effect of MetS was partly independent from the effect of several determinants of LV mass [29]. We found that both in men and women MetS was associated with a similar increase in LVH/h^2.7^ prevalence and a similar relative risk for LVH/h^2.7^. However, the study sample size was not planned to test specific gender differences in MetS and LVH relationship and therefore it is not possible to confirm a previous report [29].

In our population, as well as in larger populations such as the one of the PAMELA study [30], patients with MetS had significantly higher BP. Higher SBP, particularly through increased cardiac load, can partially explain the increased LVM found in MetS subjects. However, in our logistic regression models, MetS maintained its relationship with LVH independently of SBP, even if SBP was the only variable independently correlated to LVH when all different components of MetS were accounted for.

Moreover, patients with MetS had significantly higher eGFR and 5-fold higher ACR, indicating that in these hypertensive patients the kidneys too showed signs of increased overload and damage. Therefore in hypertensive patients the presence of MetS associates with more severe organ damage and higher cardiovascular risk.

However, it is still unclear if MetS, even across different definitions, adds something more than the sum of each of its components in predicting organ damage. In our study MetS was not able to predict LVH independently of BMI, the most used, widespread and clinically useful marker of increased adiposity. Although waist, as a single component, was not an independent determinant of LVH in our analysis, there is increasing evidence that regional fat distribution (abdominal but even epicardial, pericardial or mediastinal adipose tissue) could contribute to cardiac remodeling and hypertrophy [31], [32]. The effect of MetS on the relationship among fat mass, fat distribution and LVM is however still unclear. When the different components of MetS were studied in logistic regression models along with BMI, only SBP and BMI resulted in having an independent relationship with LVH. Some authors reported that MetS is a risk factor for LVH independently of BMI [26], [33], and might play an important role in cardiac restructure above BP and increased BMI. However, several differences must be considered between the present study and previous published papers. First, population, inclusion and exclusion criteria, as well as criteria used to define MetS were different. Our population excluded elderly and people with severe reduction of eGFR to limit as much as possible confounding factors, such as vascular damage and severe renal impairment. Second, in both published papers [26], [33], 65% and 55% of the studied patients, respectively, were on pharmacological treatment that was discontinued 2 weeks before enrollment. This approach obviously led to a return of BP towards original levels without a similar regression in LVM, an important confounding factor that we think is often overlooked. This confounding factor is particularly important when the population is mixed regarding treatment and when a considerable percentage was not treated at all, as in previous studies [26], [33]. Studies with never treated hypertensive patients are very rare but are source of very important information [29]. In our study, we chose not to suspend treatment and, on the contrary, we enrolled only untreated patients or patients on stable anti-hypertensive therapy in order to have the best possible “real” correspondence between obtained BP measurements, LVM, and pharmacological treatment. Third, we preferred to use different statistical methods (logistic regression rather than multiple linear regression analysis with MetS as a dummy variable). We believe that these differences in methods overall improved the study and led to our results.

Our data are also supported, at least in part, by some previous published investigations. For example, a recent paper by Tsioufis et al. [34], demonstrated that MetS did not worsen hypertension-induced restructuring of left ventricle and large arteries in untreated hypertensive patients. Moreover, in their multiple linear regression model, in which MetS was included as a dummy variable together with age, sex, BMI, smoking, 24-h SBP and DBP, only BMI, age, and 24-h SBP resulted independent predictors of LVM/h^2.7^ [34]. Indeed we performed a logistic regression analysis using LVH/h^2.7^ as the dependent variable, similar predictors resulted also in the subgroup with 24-hour SBP (table 4, model 3 and 4). Moreover, the significant difference and relevance of SBP over DBP underlines the key importance of volume-related BP increase, another characteristic of Ow/Ob related hypertension.

Some limitations of this study must be taken into account. First, our population is made exclusively of patients studied in a single Hypertension Centre because of not completely controlled BP as referred by their general practitioners. This selection “bias” can explain the high prevalence of MetS (65%), diabetes (23.9%) and dyslipidemia (77.1%) as well as the high BP levels despite active pharmacological treatment in the vast majority of patients. It is well known that in obese hypertensive patients it is very difficult to reach BP control [8]. Thus, our study is relevant for an important condition commonly found in “real life” medical practice. Due to inclusion criteria, the mean BMI in our population is quite high (31.97 kg/m^2^) and that could, at least in part, explain the high prevalence of eccentric hypertrophy. In fact it is known that the eccentric pattern of remodeling and hypertrophy is the most prevalent in obesity, being obesity a strong predictor of eccentric LVH [35]. Moreover, increased cardiac workload because of not completely controlled hypertension may explain the higher prevalence of concentric hypertrophy in our sample as compared to other populations, such as the one of the LIFE study [36]. It is indeed well known that different patterns of LV remodeling can be observed in obesity [37]. Second, although inclusion of TIS and drug-class treatment as a dummy variable did not significantly change the results, we were not able to take into account all the details of all the different classes and subtypes of drugs assumed by the patients, which in turn might have affected the results obtained. However, this is a common limitation of many published works when “real practice” patients are studied.

In conclusion, our finding suggests that the higher prevalence of LVH and CR is strongly associated with higher BMI and SBP in Ow/Ob hypertensive patients. Increased relative risk of LVH due to the presence of MetS, once excluded age and BP, seems mediated mainly by BMI (as an index of increased adiposity), which works as both a hemodynamic and non-hemodynamic factor [6], [7]. Therefore if we consider increased adiposity as an integral part of MetS, MetS is indeed an independent risk factor for increased LVM and increased risk of LVH and CR. Otherwise, because BMI is not formally a component of the MetS definition we may conclude that MetS is not an independent risk factor because its effects are mainly mediated by increased BMI in hypertensive patients.

At the end, excessive, inappropriate adiposity, in the context of hypertension, is the key factor further influencing left ventricular mass and geometry.
